# Response of Red Blood Cell Folate to Supplementation in Nonpregnant Women is Predictable: A Proposal for Personalized Supplementation

**DOI:** 10.1002/mnfr.201700537

**Published:** 2018-01-22

**Authors:** Rima Obeid, Christiane Schön, Manfred Wilhelm, Rajiv P. Shrestha, Stefan Pilz, Klaus Pietrzik

**Affiliations:** ^1^ Department of Clinical Chemistry Saarland University Hospital Homburg Germany; ^2^ Aarhus Institute of Advanced Studies University of Aarhus Aarhus C Denmark; ^3^ BioTeSys GmbH Esslingen Germany; ^4^ Department of Mathematics, Natural and Economic Sciences University of Applied Sciences Ulm Ulm Germany; ^5^ Octet Research Inc. Boston MA USA; ^6^ Division of Endocrinology and Diabetology Department of Internal Medicine Medical University of Graz Graz Austria; ^7^ Department of Nutrition and Food Science Rheinische Friedrich‐Wilhelms University Bonn Germany

**Keywords:** birth defects, folate requirement, pregnancy, RBC‐folate, supplementation

## Abstract

**Scope:**

We modeled red blood cell (RBC)‐folate response to supplementation and developed personalized folate supplementation concepts.

**Methods and results:**

The changes of RBC‐folate were modeled in a time‐ (4 or 8 weeks) and dose‐ (400 or 800 μg d^−1^ folate) dependent manner. Post‐supplementation RBC‐folate levels were predicted from folate‐loading capacities (= measured RBC‐folate – [baseline RBC‐folate × RBC‐survival]). The prediction equations were validated in 119 participants. The median increase of RBC‐folate was higher in the 800 μg d^−1^ than in the 400 μg d^−1^ group (275 vs 169 nmol L^−1^ after 4 weeks, and 551 vs 346 nmol L^−1^ after 8 weeks). Medians (interquartile range) of RBC‐folate loading were (4 weeks: 299 (160) vs 409 (237) nmol L^−1^) and (8 weeks: 630 (134) versus 795 (187) nmol L^−1^) in the 400 and 800 μg d^−1^ group, respectively. The individual measured and predicted RBC‐folate values (after 4 weeks/400 μg d^−1^ = 25 + 1.27 × baseline RBC‐folate) and (after 4 weeks/800 μg d^−1^ = 65 + 1.41 × baseline RBC‐folate) did not differ significantly. The measured and predicted concentrations showed high agreement in the validation cohort.

**Conclusions:**

The models can guide nutritional recommendations in women when baseline RBC‐folate concentrations are measured and the time to pregnancy between 4 and 8 weeks.

## 1 Introduction

Prevention of folate‐responsive neural tube defects (NTDs) depends on reaching a relatively high folate status before the closure of the neural tube in the first trimester. The conventional cutoff value of red blood cell (RBC)‐folate for defining folate deficiency is <340 nmol L^–1^ (using a microbiological assay). Concentrations of RBC‐folate >906 nmol L^–1^ (measured with a microbiological assay) at early pregnancy are associated with a low risk of NTDs.^1^ A population threshold for RBC‐folate concentrations (>906 nmol L^–1^) has been recently recommended for nonpregnant women of reproductive age (strong recommendation, low quality evidence).^2^ The greatest reduction of NTDs is expected on a population level when exceeding this limit (population mean is 1000–1200 nmol L^–1^). This desirable folate range is not meant to predict the individual risk, but it can be taken as a starting point to optimize the supplementation recommendations.

Increasing blood folate to an optimal level is a challenge in women who are not exposed to mandatory fortification with folic acid. Several international organizations recommend supplementing 400–800 μg d^–1^ folic acid for women who are planning or capable of pregnancy.^3^, ^4^ No explicit recommendations exist regarding the minimal duration of supplementation before conception. The intake levels that are necessary to achieve RBC‐folate concentrations >906 nmol L^–1^ within a limited time are not known.

In Germany, a country without mandatory fortification with folic acid, the average RBC‐folate concentration in women of childbearing age is in the range between 500 and 600 nmol L^–1^.^5^, ^6^ Though the average dietary folate intake (≈200 μg d^–1^)^7^ might be sufficient to prevent severe deficiency in the majority of women from this population, it is not sufficient to prevent NTDs.

Achieving desirable RBC‐folate concentrations is determined by folate dose, duration of supplementation,^5^, ^8^, ^9^ and baseline folate status.^10^ Currently, there are no personalized recommendations that take all of these factors into consideration, especially in women not exposed to fortification. The aim of the current investigation was to establish a personalized supplementation scheme to achieve any predefined desirable RBC‐folate concentration within up to 8 weeks, provided that only baseline RBC‐folate concentrations are measured. Here, we used data on RBC‐folate concentrations from 172 young women before and after they had received 400 or 800 μg d^–1^ folate as part of a multimicronutrient supplement for a total duration of 8 weeks.^11^ Models for predicting post‐intervention RBC‐folate concentrations were developed based on the response to supplementation during the first 4 weeks interval. The RBC‐folate predictive models were then validated in a subgroup of the women in the second 4 weeks interval of the intervention. We demonstrated the calculations using 906 nmol L^–1^ as a threshold, but other values can be used by adapting similar equations. The complete study plan is shown in Supporting Information Figure S1.

## 2 Experimental Section

### 2.1 Study Setting, Participants, and Design

The study design and setting has been described elsewhere.^11^, ^12^ Briefly, a randomized open labeled controlled study was conducted between January and May 2016 in Germany (BioTeSys GmbH, Esslingen am Neckar). Women of childbearing age were randomized to receive multimicronutrient supplements containing 400 or 800 μg d^–1^ folate (Supporting Information Figure S2). The primary outcome of the original study was changes of serum 25(OH)‐vitamin D concentrations following supplementation, while changes of plasma total homocysteine (tHcy), serum folate, and RBC‐folate were secondary outcomes.

Healthy nonpregnant women aged between 18 and 45 years were invited to participate through announcements in local newspapers and Media. Exclusion criteria included taking supplements containing folate or vitamin D during the last 2 months, vascular diseases, any disease that affects intestinal absorption, cancer, diabetes, and BMI < 17 kg m^–2^ or >30 kg m^–2^.

Women who met the inclusion and none of the exclusion criteria and were willing to participate were randomized and started the intervention (*n* = 100 in the 400 μg group and *n* = 101 in the 800 μg group). The randomization was performed at BioTeSys by using the software Randlist.exe (DatInf GmbH, Tübingen, Germany). For the specific question of the present report, data analyses included the 172 women with baseline RBC‐folate concentrations <906 nmol L^–1^ and complete results from three study visits. Women with baseline RBC‐folate concentrations ≥906 nmol L^–1^ were not included in the present evaluation (*n* = 24). Information on baseline dietary folate intake was collected by using 3‐d food protocol. Folate intake was re‐estimated after 8 weeks (visit 3).

The compositions of the multimicronutrient preparations are shown in Supporting Information Table S1. The study supplements were Elevit®gynvital (Bayer Vital GmbH, Leverkusen, Germany) and Femibion®1 (Merck Selbstmedikation GmbH, Darmstadt, Germany) that contain 400 and 800 μg folate (folic acid and (6S)‐5‐CH_3_‐H_4_folate‐Ca (1:1) ), respectively. Compliance with the study supplementation was documented through counting the pills at each visit, in addition to recording the intake in a study diary.

The ethical principles for medical research involving human subjects stated in Helsinki Declaration were considered. The medical ethics commission of the Baden‐Württemberg region has reviewed and approved the study (approval number: F‐2015‐102) and all participants signed consent.

### 2.2 Blood Samples and Biochemical Assays

Fasting blood samples (≥10 h) were collected at baseline (visit 1), week 4 (visit 2), and week 8 (visit 3). Participants had to abstain from taking the supplements 24 h before the blood collection (visits 2 and 3). Serum and K^+^EDTA blood were centrifuged and separated within 30 min of blood collection. For the measurement of RBC‐folate, whole blood hemolysates were immediately prepared by diluting K^+^EDTA‐whole blood with 0.5% ascorbic acid and incubation for 3 h at ambient temperature. Serum, EDTA plasma, and blood hemolysates were immediately frozen at –70 °C until analyses of the biomarkers. Samples from the same participant were measured in the same run. All markers were measured using aliquots that were not thawed before.

Concentrations of folate in serum‐ and blood hemolysates were measured by using Chemiluminescence immunoassay (IMMULITEۚ). The concentrations of RBC‐folate were calculated by adjusting for hematocrit and serum folate measured in the same individual:

RBC-folate=( whole  blood  folate ×27)− serum  folate ×1− hematocrit ×100/ hematocrit

where 27 is the assay dilution factor. The concentrations of total homocysteine (tHcy) (i.e., folate markers) were measured in EDTA plasma by using a RP‐HPLC connected to a fluorescence detector and reagents from Chromsystems Instruments & Chemicals GmbH. The between‐day coefficients of variation (CV%) as measures of the assays performance were calculated from commercially available quality control samples. The CV% for the folate assay was 8.8% at 3.6 nmol L^–1^ and 5.2% at 24.9 nmol L^–1^. The CVs% for the tHcy assay were ≤8.2% at 10.0 and 20.6 μmol L^–1^.

### 2.3 Statistical Analyses

The distributions of the continuous variables were tested by using the Shapiro–Wilk test. The differences in normally distributed variables between any two independent groups were tested by using Student's *t*‐test for variables with normal distribution and the Wilcoxon rank sum test for shifted variables (serum and RBC‐folate and plasma tHcy). Chi‐square test was used to compare categorical variables. Wilcoxon signed rank test was used for paired comparisons of two continuous variables. Results of the continuous variables are expressed as median (25^th^–75^th^) percentiles and interquartile range (IQR) when necessary. The changes of the concentrations of the blood markers were calculated as post‐ minus pre‐intervention concentrations. The differences in the concentrations were calculated for the first interval after 4 weeks (visit 2 – visit 1), the second interval after an additional 4 weeks (visit 3 – visit 2), and for the whole duration of 8 weeks (visit 3 – visit 1). Data analyses were performed using SPSS version 24.0. For comparisons of the calculated and predicted RBC‐folate, we applied Deming regression, to generate “prediction” equations to make predicted and measured RBC‐folate concentrations comparable.^13^, ^14^ Deming regression is commonly used for method comparison for fitting a straight line to 2D data where both variables, X and Y, are measured with error. Deming regression and calculated Lin's concordance correlation coefficient were applied by using R version 3.3.1 (with libraries “deming” and “epiR”). All statistical tests were two‐sided and *p*‐values < 0.05 were considered statistically significant.

### 2.4 Modeling Changes of RBC‐Folate and Predicting RBC‐Folate

We first estimated the amount of folate that was loaded into newly formed RBCs (RBC‐loading capacity) from the two supplemental folate doses after 4 and 8 weeks. The calculations were based on the following assumptions: first, RBC survival is linear within 8 weeks; second, RBC‐production rate is constant; third, mature RBCs do not exchange their folate content with plasma folate and keep their folate content until the end of their lifespan; and fourth, folate from supplements can be loaded only to the newly synthesized RBCs. Dietary folate intake remained stable in both study groups over the 8 weeks [mean (SD) in the 172 women = 311 (130) μg d^–1^ at baseline and 294 (137) μg d^–1^ after 8 weeks; *p* = 0.08]. Thus, the post supplementation loading capacities from baseline are judged to be caused by the supplements on top of dietary folate.

The total folate amount (nmol) that was loaded per 1 litre RBCs in the first 4 weeks is:

$$\begin{array}{ccc} \text{RBC-folate}\;\text{loaded} & = & \text{RBC-folate}\;\text{measured}\;\text{at}\;\text{4}\;\text{weeks}\\ & & -\;(\text{baseline}\;\text{RBC-folate}\times 0.76) \end{array}$$

where 0.76 is the rate of RBCs that were not replaced within 4 weeks (i.e., the 28 day's survival fraction) according to a recently published model of RBC lifespan^15^ (Supporting Information Figure S3, Supporting Information Table S2). In the first 4 weeks, 24% of RBCs are assumed to be loaded from the supplemented dose of folate plus the dietary folate.

The total folate amount (nmol) that was loaded per 1 litre RBC after 8 weeks is:

$$\begin{array}{ccc} \text{RBC-folate}\;\text{loaded} & = & \text{RBC-folate}\;\text{measured}\;\text{at}\;\text{8}\;\text{weeks}\\ & & -\;(\text{baseline}\;\text{RBC-folate}\times 0.52) \end{array}$$

where 0.52 is the rate of RBCs that were not replaced within 8 weeks (i.e., 56 days)^15^ (Supporting Information Figure S3, Supporting Information Table S2). During 8 weeks, 48% of RBCs are assumed to be replaced or loaded from the supplemental folate plus the dietary folate.

Using the loading models described above, we calculated the 4‐ and 8‐weeks individual RBC‐folate loading capacities for each participating woman. In addition, the median and IQR values of RBC‐folate loading were used to construct four different formulas to predict RBC‐folate concentrations after supplementing 400 or 800 μg d^–1^ folate, provided that baseline RBC‐folate concentrations are measured and the supplements will be used for 4 or 8 weeks.

The post‐intervention measured and predicted RBC‐folate concentrations were compared after 4 or 8 weeks and four final prediction equations were developed by using Deming regression.^13^, ^14^

### 2.5 Loading RBC‐Folate Under Stationary Conditions

The study did not contain a placebo group. However, since mean dietary folate intakes remained stable between visit 1 and visit 3, RBC‐folate is expected to remain stable if the women would have not received the supplements. Under this theoretical stationary condition, the diet will also cause loading of RBCs with dietary folate that maintains a stable RBC‐folate. The individual stationary RBC‐folate load (from dietary folate) was calculated for all women:

StationaryRBC-folateload4 weeks =baselineRBC-folate−(baselineRBC-folate×0.76)

StationaryRBC-folateload8 weeks =baselineRBC-folate−(baselineRBC-folate×0.52)

After calculating the total‐ and the stationary‐RBC‐folate loads, we calculated the “net RBC‐folate load” from supplemental folate by abstracting the stationary load from the total load for each participant. For developing the final prediction equations, we used the total RBC‐folate load, because the supplementation recommendations imply maintaining at least the same dietary intake of folate as before the supplementation.

## 3 Results

Supporting Information Table S3 shows the main characteristics of the study population according to the intervention groups. The two groups did not differ in age, BMI, renal, or liver function markers. Also the distribution of users of anticontraceptives, smokers, and education levels did not differ between the groups.

### 3.1 Changes of Folate Markers After Supplementation

Concentrations of RBC‐folate, serum folate, and plasma tHcy did not differ between the study groups at baseline (**Table** 1). After 4 and 8 weeks of the intervention, women in the 800 μg d^–1^ group had higher RBC‐ and serum folate than those in the 400 μg d^–1^ group. The concentrations of tHcy were significantly lower in the 800 μg d^–1^ compared with the 400 μg d^–1^ group after 4 weeks (median 5.6 vs 6.0 μmol L^–1^, *p* = 0.013), and tended to be lower in the 800 μg d^–1^ group after 8 weeks (5.5 vs 5.9 μmol L^–1^; *p* = 0.085). The changes of RBC‐folate concentrations from baseline were significantly higher in the 800 μg d^–1^ compared with the 400 μg d^–1^ group at 4 or 8 weeks (Table 1).

**Table 1:** Concentrations of folate biomarkers measured in 172 nonpregnant women at visits 1, 2, and 3, and their changes according to allocation to 400 and 800 μg d^–1^ folate

	Visit 1 (baseline)		*p* a	Visit 2 (4 weeks)		*p* a	Visit 3 (8 weeks)		*p* a
	400 μg d^–1^	800 μg d^–1^		400 μg d^–1^	800 μg d^–1^		400 μg d^–1^	800 μg d^–1^	
*n*	88	84		88	84		88	84	
RBC‐folate, nmol L^–1^	558 (404, 707)	546 (436, 689)	0.915	694 (560, 898)	841 (677, 989)	<0.001	914 (727, 1118)	1103 (979, 1230)	<0.001
Serum folate, nmol L^–1^	13.8 (10.7, 20.3)	14.3 (12.1, 19.0)	0.541	34.6 (25.2, 41.6)	49.9 (37.6, 65.9)	<0.001	44.5 (34.9, 51.1)	67.8 (49.5, 80.4)	<0.001
tHcy, μmol L^–1^	7.3 (6.2, 8.9)	7.3 (5.8, 8.2)	0.238	6.0 (5.3, 7.0)	5.6 (4.5, 6.6)	0.013	5.9 (5.0, 6.7)	5.5 (4.8, 6.5)	0.085

	Changes visit 1 to 2 (first 4 weeks)^b^		P^c^	Changes visit 2 to 3 (next 4 weeks)^b^		P^c^	Changes visit 1 to 3 (all 8 weeks)^b^		P^c^
	400 μg d^–1^	800 μg d^–1^		400 μg d^–1^	800 μg d^–1^		400 μg d^–1^	800 μg d^–1^	
RBC‐folate, nmol L^–1^	169 (111, 245)	275 (209, 353)	<0.001	181 (98, 271)	242 (158, 360)	0.003	346 (271, 461)	551 (410, 638)	<0.001
Serum folate, nmol L^–1^	18.7 (11.6, 25.8)	32.6 (25.2, 48.7)	<0.001	9.0 (4.5, 17.4)	14.0 (1.6, 28.6)	0.067	29.1 (21.9, 35.5)	49.6 (34.8, 64.0)	<0.001
tHcy, μmol L^–1^	−1.4 (−2.2, −0.6)	−1.6 (−2.3, −0.7)	0.334	−0.3 (−0.5, 0.2)	−0.2 (−0.6, 0.4)	0.328	−1.6 (−2.4, −0.9)	−1.5 (−2.6, −0.9)	0.886

Results are shown as median (25^th^, 75^th^ percentiles). All women had baseline folate concentrations < 906 nmol L^–1^.

a p values are according to Wilcoxon rank sum test (for continuous variables).

b Changes are calculated as concentrations at 4 weeks (or 8 weeks) minus those at baseline.

c p values for the difference in the change between the study groups are according to Wilcoxon rank sum test.

RBC, red blood cell; tHcy, total homocysteine

### 3.2 RBC‐Folate‐Loading Capacity in Relation to Folate Intake and Duration of the Intervention

We defined and calculated the amount of folate loaded into RBCs as a function of a stable dietary folate, the supplemented folate dose (400 or 800 μg d^–1^), and the duration of supplementation (4 or 8 weeks) (**Table** 2). The calculated median stationary folate load under stable dietary intakes and no supplementation was 131–134 nmol L^–1^ after 4 weeks and twice of this amount after 8 weeks (262–268 nmol L^–1^) in each study group. After the first 4 weeks (visit 1 → visit 2), the median total RBC‐folate‐load (from diet plus supplements) was 299 nmol L^–1^ in the 400 μg d^–1^ group and 409 nmol L^–1^ in the 800 μg d^–1^ group (difference = 110 nmol L^–1^, *p* < 0.001). At the end of the 8 weeks (visit 1 → visit 3), the difference in total RBC‐folate loading capacity between the study groups was 165 nmol L^–1^ which corresponds to 26% difference (median 630 nmol L^–1^ vs 795 nmol L^–1^). The median total RBC‐folate load was 94–111% higher at the end of the 8 weeks compared with the first 4 weeks (median 299 vs 630 nmol L^–1^ (111%) in the 400 μg d^–1^; 409 nmol L^–1^ vs 795 nmol L^–1^ (94%) in the 800 μg d^–1^ after 4 and 8 weeks, respectively) (Table 2). The median net RBC‐folate load caused by the supplements (= total folate load – stationary load) at 4 weeks was 169 nmol L^–1^ in the 400 μg d^–1^ group and 275 nmol L^–1^ in the 800 μg d^–1^ group, while the corresponding net RBC‐folate load after 8 weeks was 346 nmol L^–1^ and 551 nmol L^–1^, respectively (Table 2).

**Table 2:** RBC‐folate load without supplements (hypothetical) and according to the folate dose and intervention duration in young women who started the trial with a baseline RBC‐folate concentration < 906 nmol L^–1^ (n = 172)

Folate (nmol) load/ 1 litre RBC	Folate dose = 400 μg d^–1^	Folate dose = 800 μg d^–1^	p^h^
Number	88	84	
**Stationary folate load** a			
visits 1→ 2 (first 4 weeks)^b^	134 (97, 170)/73	131 (105, 165)/60	0.915
visits 1→ 3 (total 8 weeks)^c^	268 (194, 340)/146	262 (209, 331)/122	0.915
**Observed total folate load** d			
visits 1→ 2 (first 4 weeks)^e^	299 (236, 397)/160	409 (347, 481)/237	<0.001
visits 1→ 3 (total 8 weeks)^f^	630 (492, 729)/134	795 (711, 898)/187	<0.001
**Net folate load from supplements** g			
visits 1→ 2 (first 4 weeks)	169 (111, 245)/134	275 (209, 353)/144	<0.001
visits 1→ 3 (total 8 weeks)	346 (271, 461)/190	551 (410, 638)/228	<0.001

Data are median (25^th^, 75^th^ percentiles)/ interquartile range (IQR).

a Stationary RBC‐folate load is a hypothetical condition that is calculated under stable dietary intake and no additional supplements.

according to the equations:

b = baseline RBC‐folate ‐ (baseline RBC‐folate × 0.76);

c = baseline RBC‐folate ‐ (baseline RBC‐folate × 0.52) where 0.76 and 0.52 are the survival fraction of RBCs after 28 and 56 days according to Shrestha et al.^15^

d Observed total folate load from diet plus supplements.

according to the equations:

e = measured RBC‐folate at 4 weeks ‐ (baseline RBC‐folate × 0.76);

f = measured RBC‐folate at 8 weeks ‐ (baseline RBC‐folate × 0.52).

g Net folate load from the supplements only (= total folate load under supplements – stationary folate load).

h p values are according to Wilcoxon rank sum test.

RBC, red blood cell.

### 3.3 Predicting Post‐Supplementation RBC‐Folate from Baseline Levels (Native Models)

Since baseline RBC‐folate concentrations were measured, we used the dose‐ and duration‐specific medians of total RBC‐folate load to predict women's post‐intervention RBC‐folate concentrations (**Table** 3). Supporting Information Figures S4 and S6 show the relationship between the measured and the predicted RBC‐folate values in addition to native regression models. Supporting Information Figures S5 and S7 show the corresponding recalibrated models after applying Deming regression.

**Table 3:** Equations for prediction of post‐intervention RBC‐folate (nmol L^–1^) in women of childbearing age given that baseline RBC‐folate concentrations are measured and are below the desirable level (here 906 nmol L^–1^)

	Predicted post‐intervention RBC‐folate		
	From RBC‐folate net load (supplements only)^a^	From stationary load (diet only)	From total load (supplements plus diet)
Corresponding equations	Median net load (IQR) + (baseline RBC‐folate × RBC‐survival)^b^	Baseline RBC‐folate ‐ (baseline RBC‐folate × RBC‐survival)	Median total load (IQR)^c^ + (baseline RBC‐folate × RBC‐survival)^b^
4 weeks (visit 2 vs 1)			
400 μg d^–1^	169 (134) + (baseline RBC‐folate × 0.76)	133 (67)	299 (160) + (baseline RBC‐folate × 0.76)
800 μg d^–1^	275 (144) + (baseline RBC‐folate × 0.76)	133 (67)	409 (237) + (baseline RBC‐folate × 0.76)
8 weeks (visit 3 vs 1)			
400 μg d^–1^	346 (190) + (baseline RBC‐folate × 0.52)	265 (134)	630 (134) + (baseline RBC‐folate × 0.52)
800 μg d^–1^	551 (228) + (baseline RBC‐folate × 0.52)	265 (134)	795 (187) + (baseline RBC‐folate × 0.52)

a The equations are based on median (IQR) of net RBC‐folate load from supplements only (total load – stationary load) as shown in Table 2.

b The RBCs survival fractions are 0.76 and 0.52 after 4 and 8 weeks, respectively.

c Total load levels are according to the equations: = measured RBC‐folate at 4 weeks ‐ (baseline RBC‐folate × 0.76); or = measured RBC‐folate at 8 weeks ‐ (baseline RBC‐folate × 0.52) (details in Table 2).

IQR, interquartile range; RBC, red blood cell.

### 3.4 Comparisons of Measured and Predicted Post‐Supplementation RBC‐Folate

**Table** 4 shows the recalibrated final equations of predicted RBC‐folate in addition to the results of Deming regression after recalibrations (Lin's concordance correlation, intercepts, and slope of the regression). The recalibrated equations (at 4 and 8 weeks) were then applied to individual baseline concentrations to calculate post‐intervention RBC‐folate from the measured baseline RBC‐folate concentrations (**Table** 5). The predicted and measured post‐supplementation RBC‐folate showed a perfect match around the mean. However, the relationship between the two variables showed a slope < 1 and an intercept > 0, thus indicating a systematic bias that was found to be concentration‐ and time‐dependent (Supporting Information Figures S4–S6). The difference between the measured and the predicted RBC‐folate was negative (predicted > measured) at low baseline RBC‐folate concentrations and positive (predicted < measured) at higher baseline RBC‐folate (Table 5). The recalibrated intercepts and slopes were not significantly different from the ideal correlations (Supporting Information figures S5 and S7 and Table 4). After correction for the systematic bias and recalibration we got the final prediction equations for post‐intervention RBC‐folate concentrations (Table 4):

$$\begin{array}{ccc} & & \text{RBC-folate}\;\left(\text{nmol}\;L^{-1}\right)\;\text{after}\;\text{4}\;\text{weeks}\;\text{on}\;400\;\mu gd^{-1}\\ & & \;=25+1.27\times \;\text{baseline}\;\text{RBC-folate}. \end{array}$$

RBC-folate( nmol L−1) after 4 weeks  on 800μgd−1=65+1.41×baselineRBC-folate

**Table 4:** Results of Deming regression^a^ relating predicted RBC‐folate to measured RBC‐folate after supplementation

Duration and dose	Lin's concordance correlation (95% CI)	Intercept (95% CI)	Slope (95% CI)	Final prediction equations after applying Deming regression and Lin's correction
**4 weeks (visit 2 vs 1)**				
400 μg d^–1^, n = 88	0.848 (0.778, 0.897)	−66 (−169, 37)	1.10 (0.95, 1.24)	25 + 1.27 × baseline RBC‐folate
800 μg d^–1^, n = 84	0.771 (0.670, 0.844)	−150 (−386, 85)	1.19 (0.90, 1.48)	65 + 1.41 × baseline RBC‐folate
**8 weeks (visit 3 vs 1)**				
400 μg d^–1^, n = 88^b^	0.591 (0.494, 0.674)	−390 (−703, −77)	1.43 (1.07, 1.79)	−100 + 1.86 × baseline RBC‐folate
800 μg d^–1^, n = 84	0.497 (0.387, 0.594)	−619 (−1455, 218)	1.55 (0.76, 2.34)	83 + 1.79 × baseline RBC‐folate

a Deming‐regression has shown that the measured RBC‐folate and the one predicted using the uncorrected equations had an intercept that was significantly greater than 0 and a slope that was significantly lower than 1. Deming regression applied after recalibration showed that the intercept was not significantly different from 0 and the slope was not significantly different from 1 for the first 4 weeks period.

b When using this calibrated equation for 400 μg d^–1^ and 8 weeks, the predicted concentrations remained different from the measured concentrations.

CI, confidence intervals; RBC, red blood cells.

**Table 5:** Measured and predicted concentrations of post‐supplementation RBC‐folate before and after recalibration using Deming regression^a^

	Post‐supplementation RBC‐folate, nmol L^–1^				
Duration and dose	Measured concentrations^b^^,^ f	Predicted from total loading models^b^^,^ c	Difference Measured – native predicted [Mean, SD (min,max)]	p^d^	Predicted after applying the corrected equations^b^^,^ e
4 weeks (visit 2 vs 1)					
400 μg d^–1^	694 (560, 898)	723 (606, 837)	[9, 118 (–311, 324)]	0.632	734 (538, 923)
800 μg d^–1^	841 (677, 989)	824 (740, 933)	[13, 131 (–273, 532)]	0.758	836 (679, 1037)
8 weeks (visit 3 vs 1)					
400 μg d^–1^	914 (727, 1118)	920 (840, 998)	[13, 196 (–342, 784)]	0.999	938 (652, 1216)
800 μg d^–1^	1103 (979, 1230)	1079 (1022, 1153)	[17, 172 (–395, 938)]	0.524	1061 (863, 1316)

a The study included women who started the trial with baseline RBC‐folate concentrations below 906 nmol L^–1^; n = 88 in the 400 μg d^–1^ group and n = 84 in the 800 μg d^–1^ group.

b Results are shown as median (25^th^, 75^th^ percentiles).

c Predicted RBC‐folate is according to the uncorrected native formulas for total load as shown in Table 3.

d P values between the measured and the predicted RBC‐folate are according to Wilcoxon signed rank test. P values for comparisons of the medians were not significant. However, the predicted data showed a systematic error (slope < 1) at 4 and a larger error at 8 weeks.

e Predicted RBC‐folate corrected for the systematic error using Deming regression and recalibration according to the final equations shown in Table 4.

f RBC‐folate concentrations were measured by using IMMULITE.

RBC, red blood cell; SD, standard deviation.

After 8 weeks of supplementation (visit 3 vs 1), the systematic bias between the predicted and the measured RBC‐folate was corrected after recalibration in the 800 μg d^–1^ group, but the difference remained significant in the 400 μg d^–1^ group (Supporting Information Figure S7).

### 3.5 Internal and External Validation of the Final Personalized Equations

**Figure 1:**
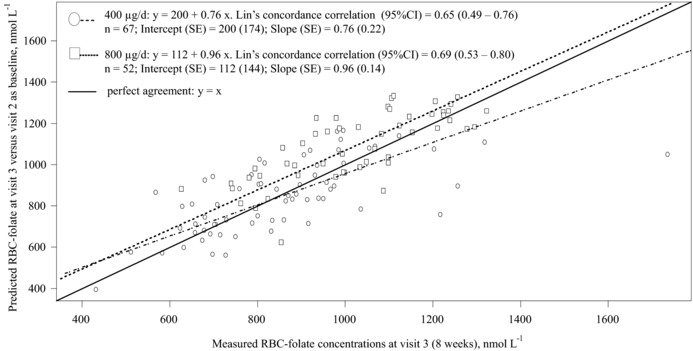
Deming regression and Lin's concordance correlation coefficient in the validation cohort. The predicted RBC‐folate concentrations at visit 3 (y‐axis) were compared with the concentrations measured by using IMMULITE at visit 3 (x‐axis). The predicted RBC‐folate levels at visit 3 were calculated from levels measured at visit 2 (4 weeks). The cohort that was used for validation included 67 women in the 400 μg d^–1^ group and 52 women in the 800 μg d^–1^ group. All women in the validation cohort had RBC‐folate < 906 nmol L^–1^ at visit 2 (baseline for visit 3). Predicted post‐intervention RBC‐folate for the validation cohort in the 400 μg d^–1^ group at visit 3 (total 8 weeks) = 25 + 1.27 × RBC‐folate at visit 2; Predicted post‐intervention RBC‐folate in the 800 μg d^–1^ group at visit 3 (8 weeks) = 65 + 1.41 × RBC‐folate at visit 2. The slopes and the intercepts of the associations between the measured and the predicted RBC‐folate are not significantly different from the ideal correlation for both study groups.

The final recalibrated equations (Table 4) were validated using the dataset between visits 2 and 3. RBC‐folate concentrations at visit 3 were predicted using measured RBC‐folate at visit 2 as a baseline for visit 3. The validation cohort included only women (*n* = 119) who reached visit 2 with RBC‐folate concentrations <906 nmol L^–1^ (67 women in the 400 μg d^–1^ group and 52 women in the 800 μg d^–1^ group), since this was a critical inclusion criteria in the present study (**Table** 6). Deming regression was again applied to investigate the agreement between the predicted and the measured RBC‐folate in the validation dataset (**Figure** 1). No significant differences were found between the predicted and the measured RBC‐folate. The prediction equations were additionally applied to individual data from 23 women participating in an earlier supplementation trial that used placebo or 800 μg d^–1^ folic acid^9^ and where RBC‐folate were measured using the original microbiological assay (Data not shown).^16^ The results of the measured and predicted RBC‐folate levels were not significantly different after 4 weeks [mean (SD) = 918 (225) vs 987 (239) nmol L^–1^, respectively; *p* = 0.106]. The measured levels were slightly lower than the predicted ones after 8 weeks [1166 (214) vs 1253 (303) nmol L^–1^, respectively; *p* = 0.059].

**Table 6:** Measured versus predicted RBC‐folate concentrations in the validation cohort (visit 3 versus 2) calculated by using the final equations after corrections for systematic error

		Measured RBC‐folate, nmol L^–1^		Predicted RBC‐folate, nmol L^–1^ c
The validation cohort^a^		Visit 2	Visit 3	Visit 3
Visit 2 vs 3 (8 weeks)^b^	400 μg d^–1^	639 (542, 767)	833 (698, 967)	837 (713, 999)
Visit 2 vs 3 (8 weeks)^b^	800 μg d^–1^	753 (636, 827)	1032 (887, 1151)	1126 (962, 1231)

a The validation cohort included 119 women who had RBC‐folate < 906 nmol L^–1^ at visit 2 (67 women in the 400 μg d^–1^ group and 52 women in the 800 μg d^–1^ group). RBC‐folate concentrations at visit 2 were considered as baseline levels for visit 3.

b Results are shown as median (25^th^, 75^th^ percentiles).

c Predicted RBC‐folate levels at visit 3 were calculated from RBC‐folate measured at visit 2 according to the final corrected equations: for the 400 μg d^–1^ at visit 3 (total 8 weeks) = 25 + 1.27 × RBC‐folate at visit 2; for the 800 μg d^–1^ at visit 3 (8 weeks) = 65 + 1.41 × RBC‐folate at visit 2.

RBC, red blood cell.

By applying the same concept, we estimated the lowest baseline RBC‐folate threshold that can theoretically be increased to a target concentration (i.e., 906 nmol L^–1^) within 4 weeks if 400 or 800 μg d^–1^ were to be supplemented (Supporting Information Table S4). We found that RBC‐folate concentrations close to the population mean (approximately 550 nmol L^–1^) would require 800 μg d^–1^ folate for 4 weeks or 400 μg d^–1^ for 8 weeks to reach the 906 nmol L^–1^ (Supporting Information Table S4).

The prediction equations were applied to mean values from various independent studies or to sub‐groups according to the 677 C to T polymorphism in methylenetetrahydrofolate reductase gene^5^, ^17^, ^18^, ^19^ (Supporting Information Table S5). The predicted RBC‐folate values were similar to the measured ones, despite heterogeneity of the studies (i.e., analytical methods, populations, baseline levels, and age).

## 4 Discussion

We quantified the effect of folate dose‐ and supplementation duration on the changes of RBC‐folate in young nonpregnant women. The novel results are that changes of RBC‐folate concentrations are predictable and can be used to optimize supplementation recommendations on an individual level or on a population level (i.e., based on population mean). The results are particularly relevant for women of childbearing age from countries not applying folic acid fortification in order to reach desirable RBC‐folate level within several weeks before conception. The prediction equations of post‐supplementation RBC‐folate concentrations showed high reproducibility in the validation cohort from the present study. A preliminary evaluation revealed good agreements with published results on post‐intervention measured RBC‐folate concentrations.

### 4.1 The Concept of Personalized Supplementation

The global recommendations to supplement 400–800 μg folic acid per day before pregnancy are arbitrary and not based on dose‐ and time‐response curves. We estimated that the current recommendation to supplement 400 μg folate per day for 4 weeks would be sufficient to achieve RBC‐folate of 906 nmol L^–1^ only when women's baseline RBC‐folate is ≥694 nmol L^–1^ which corresponds to the upper 25% of the population in the current study. Supplementation with 800 μg d^–1^ for ≥4 weeks (or 400 μg d^–1^ for at least 8 weeks) was necessary to raise RBC‐folate concentrations within this limited time when women's folate concentrations were close to the population average (550 nmol L^–1^). When baseline RBC‐folate are below the average, the higher dose and longer duration are needed to reach protective RBC‐folate levels. Doubling the dose (from 400 to 800 μg d^–1^) caused on average 37% higher RBC‐loading at 4 weeks while extending the supplementation duration from 4 to 8 weeks had a stronger influence (i.e., 94–111%) on RBC‐folate.

### 4.2 Validity and Generalizability of the Results

In contrast to earlier reports^19^, ^20^, ^21^ that addressed reaching a steady state upon long term supplementation, our study was concerned with short‐term RBC‐folate repletion. Earlier studies have used different kinetic models that were directly inferred from changes of total RBC‐folate over up to 24 weeks.^5^, ^20^, ^22^ Pietrzik et al. found that concentrations of RBC‐folate increased progressively in the first 8 weeks, after which the curve was expected to be flattening until reaching a steady state.^5^ The changes of RBC‐folate concentrations in our study after 8 weeks are comparable with earlier reports from the same population and similar baseline folate concentrations (Supporting Information Table S5).^5^, ^6^

We measured RBC‐folate using the IMMULITE assay, which could be argued to have influenced the prediction equations. However, the bias between folate assay methods is mostly systematic (i.e., the methods variance is constant over the measurement range). Thus, the relationship between baseline and post‐intervention levels is expected to remain constant when applying a conversion factor to the pre‐ and post‐supplementation concentrations. Additional validation of our models using the reference method for RBC‐folate assay (i.e, microbiological) may be necessary.

Our approach of estimating RBC‐folate loading capacity was based on an independent model of RBC‐lifespan.^15^ Changes of RBC‐folate loading capacity reflected folate enrichment better than total RBC‐folate concentrations. The observed systematic differences between the predicted (based on loading capacity) and the measured RBC‐folate in the native models deserves further assessment. The predicted RBC‐folate was overestimated in the low measured RBC‐folate range and underestimated in the high range. This bias could be due to different kinetics of the receptors that take up folate into RBCs or to methylenetetrahydrofolate reductase gene C677T polymorphism that is associated with lower RBC‐folate levels and lower response to supplementation.^23^ Factors that influence RBC‐folate loading upon supplementation need further evaluation.

### 4.3 Impact of the Results on Supplementation Recommendations

The results of the present study may impact supplementation recommendations for women planning or capable of pregnancy.^4^ The prediction equations can be used for revisiting the folate supplementation recommendations before pregnancy. Quantifying the response of RBC‐folate concentrations to different folate doses and intervention durations can be used for developing personalized curves for folate supplementation. For example, a woman with a baseline RBC‐folate of 377 nmol L^–1^ is expected to achieve 504 nmol L^–1^ or 597 nmol L^–1^ when using 400 or 800 μg d^–1^, respectively for 4 weeks. While the same woman can reach 758 nmol L^–1^ when using 800 μg d^–1^ for 8 weeks ( = 83 + 1.79 × baseline RBC‐folate). Additionally, women with RBC‐folate levels ≤596 nmol L^–1^ are unlikely to achieve levels >906 nmol L^–1^ within 4 weeks without taking 800 μg d^–1^ folate. Obviously, this approach requires routine measurement of baseline RBC‐folate concentrations which is currently not widely applied.

The generalizability of the data needs further investigations. We did not apply our models to individual data from other population groups (pregnant women, elderly, men, and children), individuals with low compliance, those receiving folate doses >800 μg d^–1^, or those with high baseline folate concentrations (i.e., countries applying fortification with folic acid). Furthermore, RBCs lifespan affects the predicted post‐supplementation RBC‐folate concentrations. However, RBCs lifespan and folate‐loading capacity could be affected by iron and/or vitamin B12 deficiency.^24^

In summary, the response of RBC‐folate to a given folate dose in women of childbearing age can be predicted from baseline RBC‐folate concentrations. Doubling the folate dose (from 400 to 800 μg d^–1^) caused on average 37% higher folate loading into RBCs in the first 4 weeks and 26% after 8 weeks, while extending the supplementation duration (from 4 to 8 weeks) increased the RBC‐folate load by 94 to 111%. We suggest using our models for optimizing folate supplementation recommendations on a population level and in a personalized targeting of supplementation in women who intend to achieve RBC‐folate levels above any given level (i.e., 906 nmol L^–1^) within a short time period. Factors that determine RBC‐folate loading capacity as alternative to total RBC‐folate concentrations need further investigations.

**Abbreviations**
RBC: red blood cells
NTD: neural tube defect
tHcy: total homocysteine

## Conflict of Interest

The authors have declared no conflict of interest. All authors have read and approved the final article. BioTeSys GmbH is an independent third party research institute that was responsible for designing the study, recruitment, performance, data collection, measurements, and data analyses. BioTeSys GmbH kept record of the original data and is responsible for the data integrity and final analyses. The sponsor had no role in conducting the study, the concept of the present publication, data analysis, or reporting the results.

## Supporting information

Supplementary material

Supplementary material
