# Supplementary material for: Response of Red Blood Cell Folate to Supplementation in Nonpregnant Women is Predictable: A Proposal for Personalized Supplementation
Source: Mol Nutr Food Res. 2018 Jan 22;62(4):1700537. doi: 10.1002/mnfr.201700537 (PMC5838518; doi:10.1002/mnfr.201700537)
Supplement: Supplementary file 1 — Supplementary material [file MNFR-62-na-s001.ppt]

## Slide 1
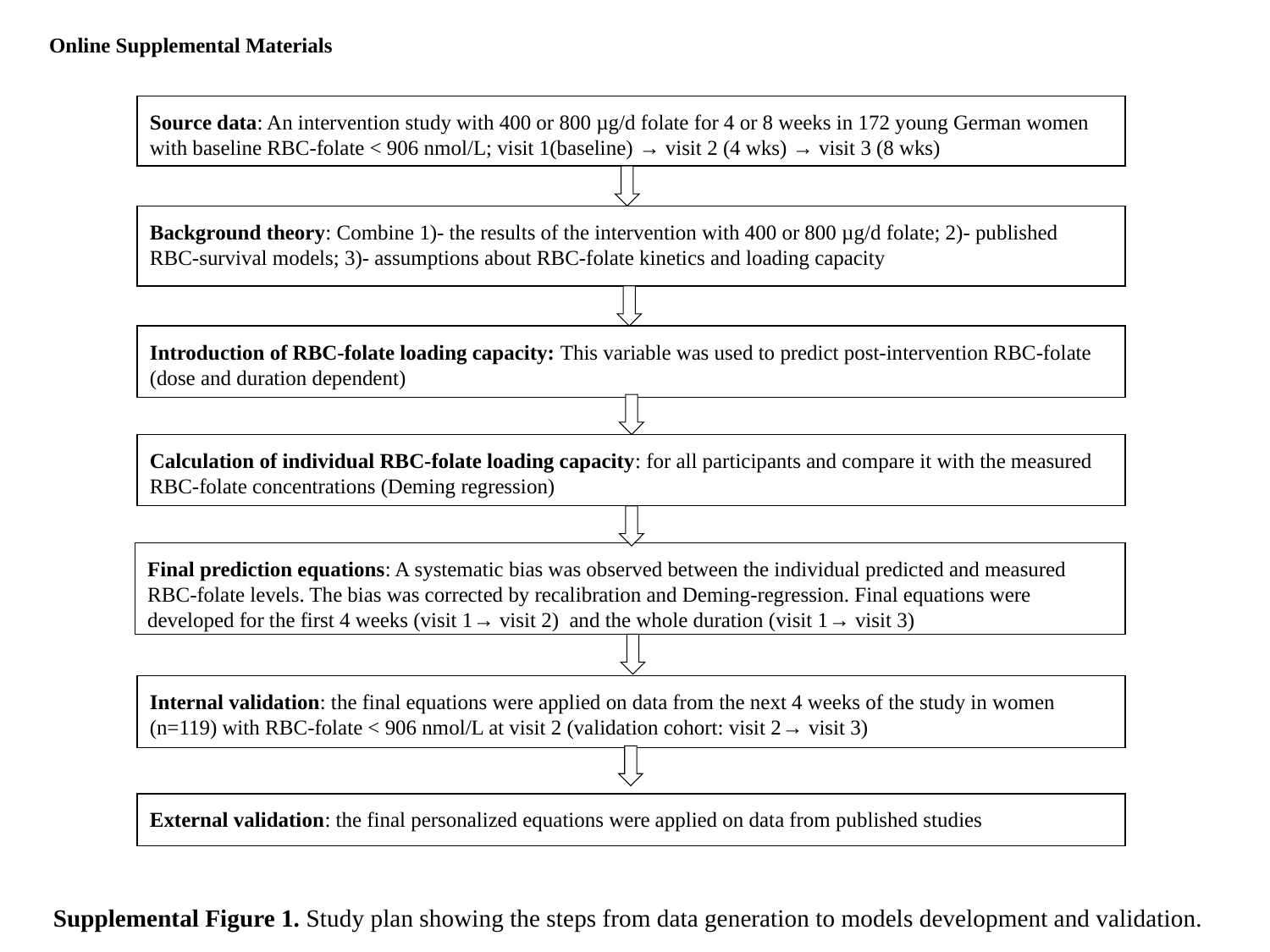

Online Supplemental Materials
Source data: An intervention study with 400 or 800 µg/d folate for 4 or 8 weeks in 172 young German women with baseline RBC-folate < 906 nmol/L; visit 1(baseline) → visit 2 (4 wks) → visit 3 (8 wks)
Background theory: Combine 1)- the results of the intervention with 400 or 800 µg/d folate; 2)- published RBC-survival models; 3)- assumptions about RBC-folate kinetics and loading capacity
Introduction of RBC-folate loading capacity: This variable was used to predict post-intervention RBC-folate (dose and duration dependent)
Calculation of individual RBC-folate loading capacity: for all participants and compare it with the measured RBC-folate concentrations (Deming regression)
Final prediction equations: A systematic bias was observed between the individual predicted and measured RBC-folate levels. The bias was corrected by recalibration and Deming-regression. Final equations were developed for the first 4 weeks (visit 1→ visit 2) and the whole duration (visit 1→ visit 3)
Internal validation: the final equations were applied on data from the next 4 weeks of the study in women (n=119) with RBC-folate < 906 nmol/L at visit 2 (validation cohort: visit 2→ visit 3)
External validation: the final personalized equations were applied on data from published studies
Supplemental Figure 1. Study plan showing the steps from data generation to models development and validation.

## Slide 2
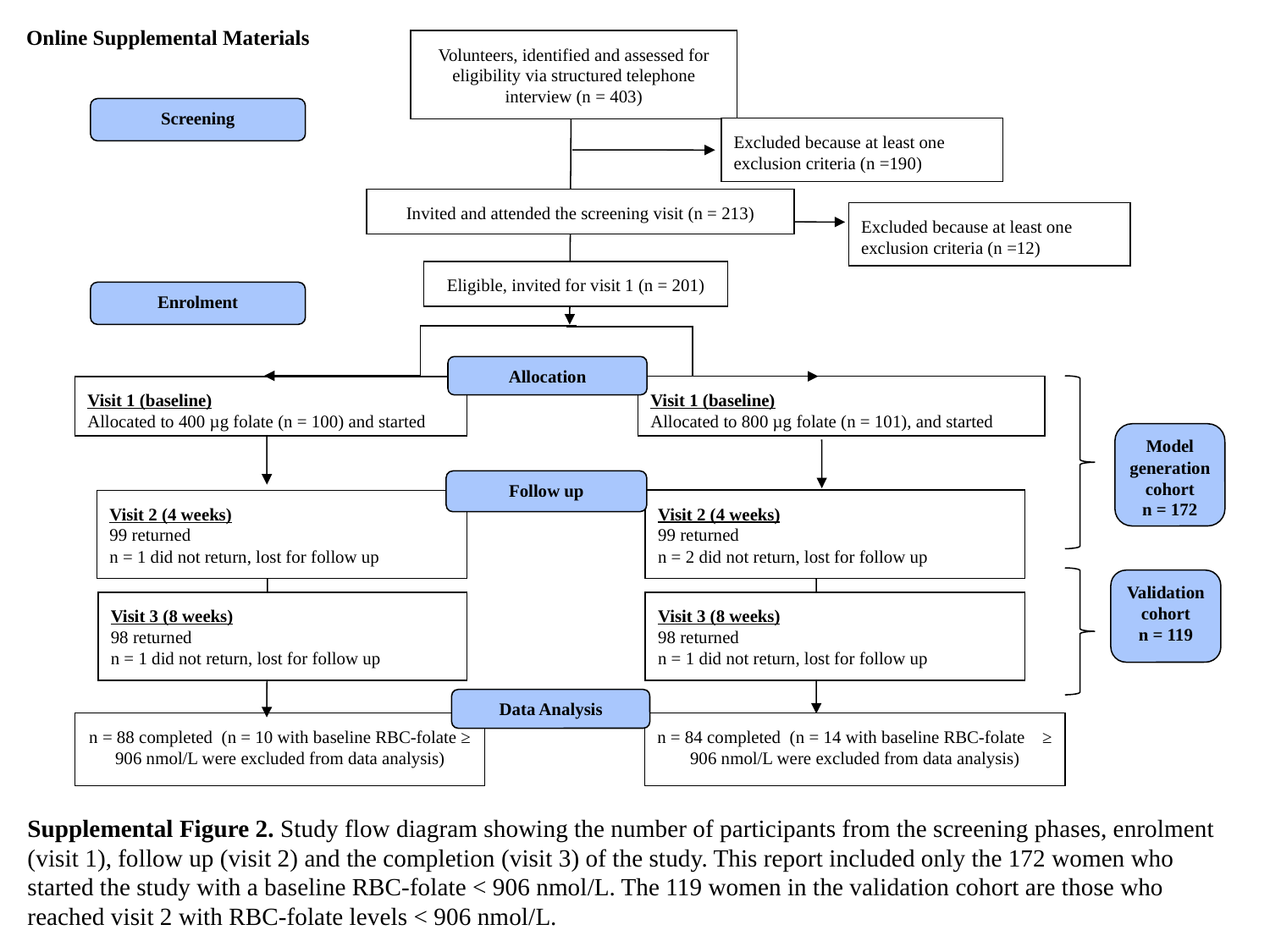

Online Supplemental Materials
Volunteers, identified and assessed for eligibility via structured telephone interview (n = 403)
Screening
Excluded because at least one exclusion criteria (n =190)
Invited and attended the screening visit (n = 213)
Excluded because at least one exclusion criteria (n =12)
Eligible, invited for visit 1 (n = 201)
Enrolment
Allocation
Visit 1 (baseline)
Allocated to 800 µg folate (n = 101), and started
Visit 1 (baseline)
Allocated to 400 µg folate (n = 100) and started
Follow up
Visit 2 (4 weeks)
99 returned
n = 2 did not return, lost for follow up
Visit 2 (4 weeks)
99 returned
n = 1 did not return, lost for follow up
Visit 3 (8 weeks)
98 returned
n = 1 did not return, lost for follow up
Visit 3 (8 weeks)
98 returned
n = 1 did not return, lost for follow up
Data Analysis
n = 88 completed (n = 10 with baseline RBC-folate ≥ 906 nmol/L were excluded from data analysis)
n = 84 completed (n = 14 with baseline RBC-folate ≥ 906 nmol/L were excluded from data analysis)
Model generation cohort
n = 172
Validation cohort
n = 119
Supplemental Figure 2. Study flow diagram showing the number of participants from the screening phases, enrolment (visit 1), follow up (visit 2) and the completion (visit 3) of the study. This report included only the 172 women who started the study with a baseline RBC-folate < 906 nmol/L. The 119 women in the validation cohort are those who reached visit 2 with RBC-folate levels < 906 nmol/L.

## Slide 3
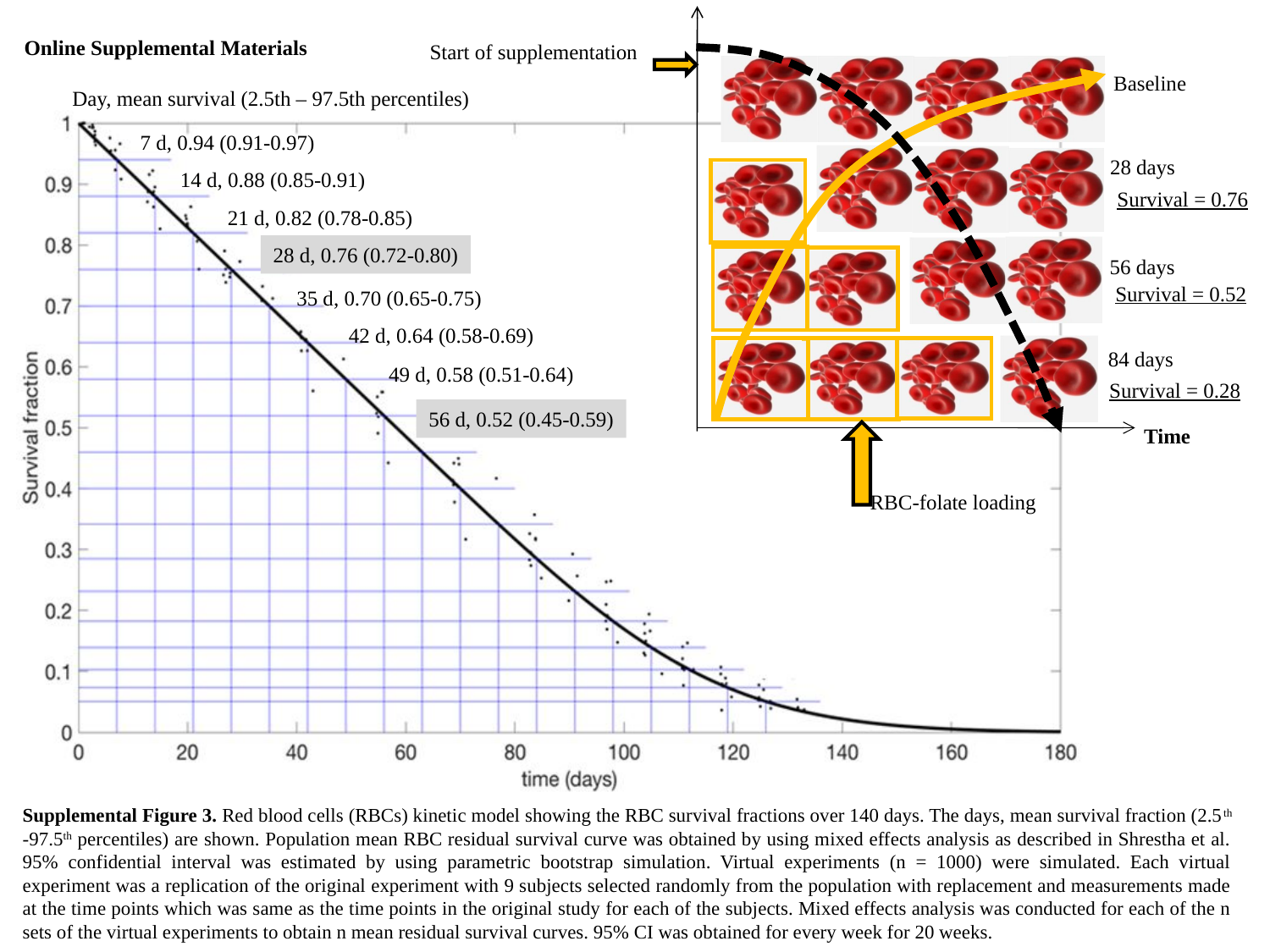

Baseline
28 days
Survival = 0.76
56 days
Survival = 0.52
84 days
Survival = 0.28
Time
Start of supplementation
RBC-folate loading
Online Supplemental Materials
Day, mean survival (2.5th – 97.5th percentiles)
7 d, 0.94 (0.91-0.97)
14 d, 0.88 (0.85-0.91)
21 d, 0.82 (0.78-0.85)
28 d, 0.76 (0.72-0.80)
35 d, 0.70 (0.65-0.75)
42 d, 0.64 (0.58-0.69)
49 d, 0.58 (0.51-0.64)
56 d, 0.52 (0.45-0.59)
Supplemental Figure 3. Red blood cells (RBCs) kinetic model showing the RBC survival fractions over 140 days. The days, mean survival fraction (2.5th -97.5th percentiles) are shown. Population mean RBC residual survival curve was obtained by using mixed effects analysis as described in Shrestha et al. 95% confidential interval was estimated by using parametric bootstrap simulation. Virtual experiments (n = 1000) were simulated. Each virtual experiment was a replication of the original experiment with 9 subjects selected randomly from the population with replacement and measurements made at the time points which was same as the time points in the original study for each of the subjects. Mixed effects analysis was conducted for each of the n sets of the virtual experiments to obtain n mean residual survival curves. 95% CI was obtained for every week for 20 weeks.

## Slide 4
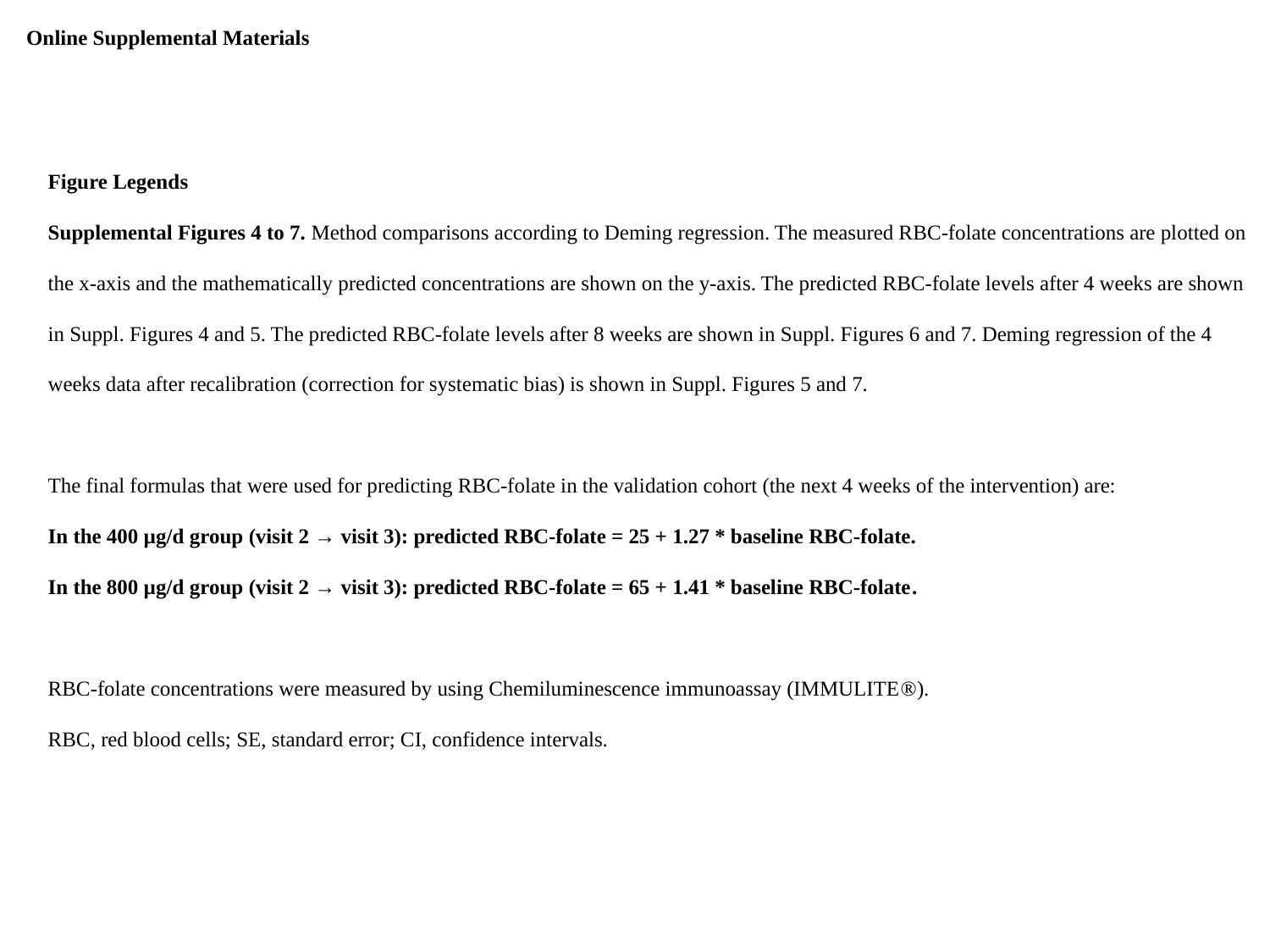

Online Supplemental Materials
Figure Legends
Supplemental Figures 4 to 7. Method comparisons according to Deming regression. The measured RBC-folate concentrations are plotted on the x-axis and the mathematically predicted concentrations are shown on the y-axis. The predicted RBC-folate levels after 4 weeks are shown in Suppl. Figures 4 and 5. The predicted RBC-folate levels after 8 weeks are shown in Suppl. Figures 6 and 7. Deming regression of the 4 weeks data after recalibration (correction for systematic bias) is shown in Suppl. Figures 5 and 7.
The final formulas that were used for predicting RBC-folate in the validation cohort (the next 4 weeks of the intervention) are:
In the 400 µg/d group (visit 2 → visit 3): predicted RBC-folate = 25 + 1.27 * baseline RBC-folate.
In the 800 µg/d group (visit 2 → visit 3): predicted RBC-folate = 65 + 1.41 * baseline RBC-folate.
RBC-folate concentrations were measured by using Chemiluminescence immunoassay (IMMULITE).
RBC, red blood cells; SE, standard error; CI, confidence intervals.

## Slide 5
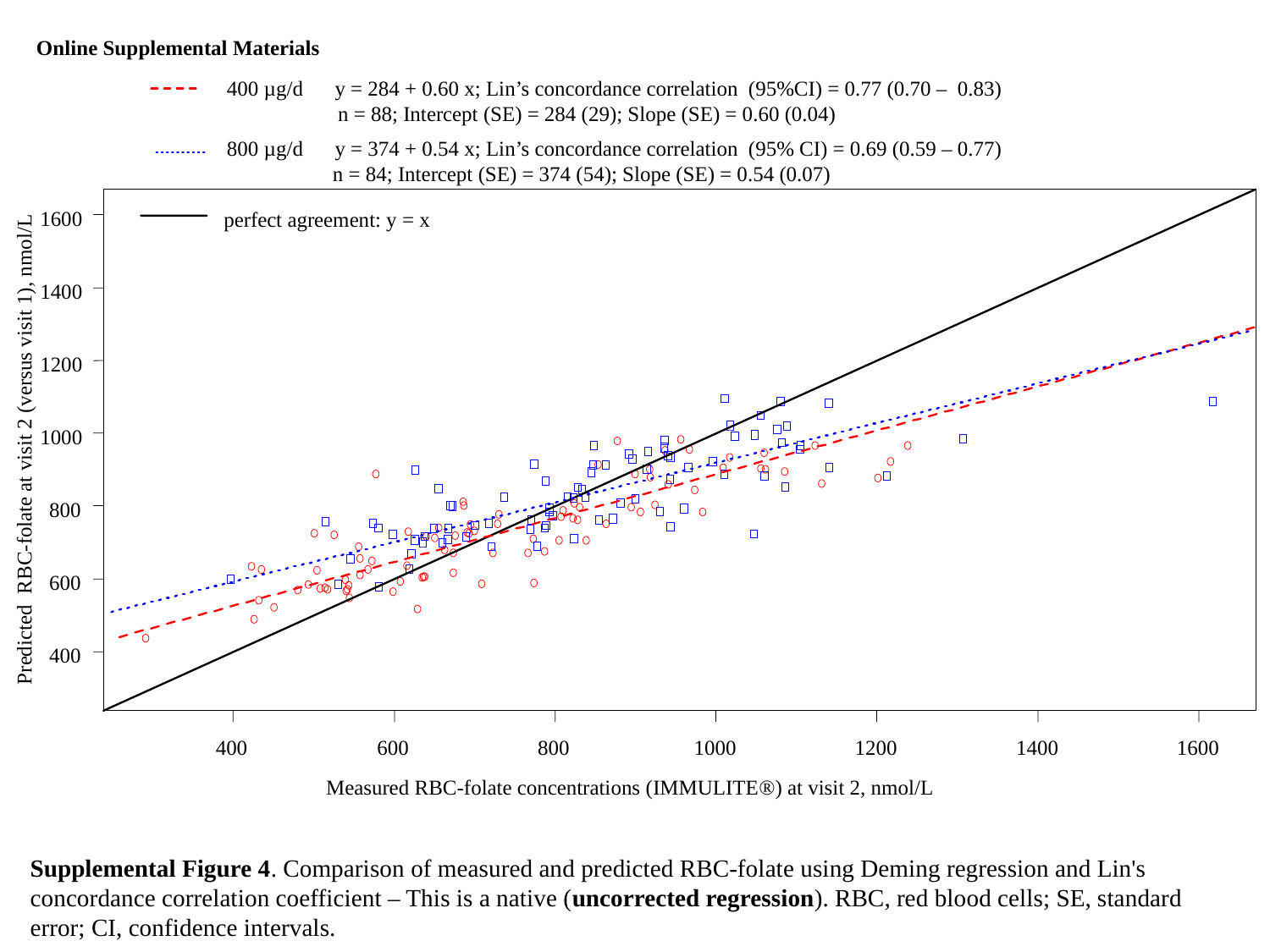

Online Supplemental Materials
400 µg/d y = 284 + 0.60 x; Lin’s concordance correlation (95%CI) = 0.77 (0.70 – 0.83)
 n = 88; Intercept (SE) = 284 (29); Slope (SE) = 0.60 (0.04)
800 µg/d y = 374 + 0.54 x; Lin’s concordance correlation (95% CI) = 0.69 (0.59 – 0.77)
 n = 84; Intercept (SE) = 374 (54); Slope (SE) = 0.54 (0.07)
1600
perfect agreement: y = x
1400
1200
1000
Predicted RBC-folate at visit 2 (versus visit 1), nmol/L
800
600
400
400
600
800
1000
1200
1400
1600
Measured RBC-folate concentrations (IMMULITE) at visit 2, nmol/L
Supplemental Figure 4. Comparison of measured and predicted RBC-folate using Deming regression and Lin's concordance correlation coefficient – This is a native (uncorrected regression). RBC, red blood cells; SE, standard error; CI, confidence intervals.

## Slide 6
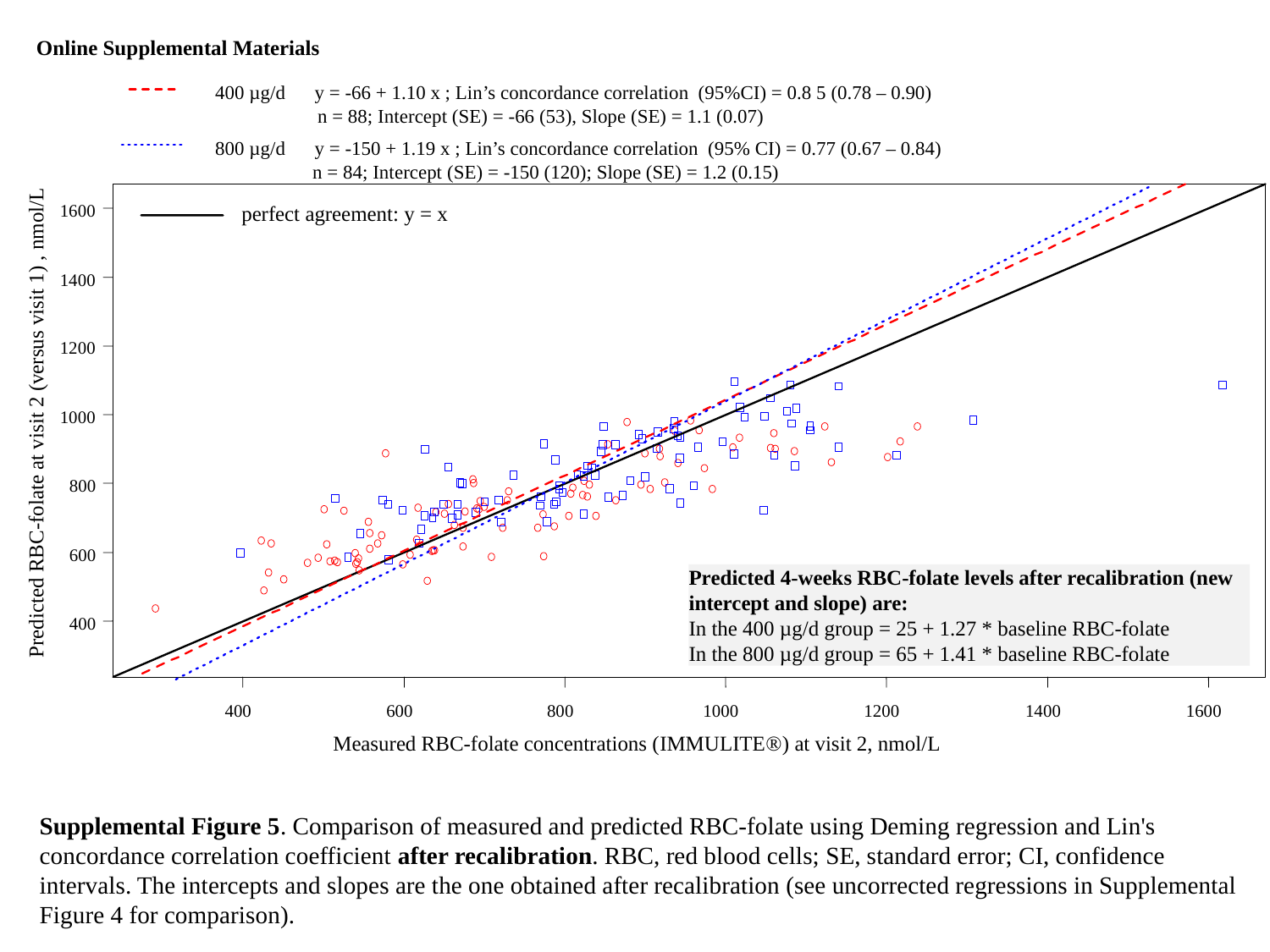

Online Supplemental Materials
400 µg/d y = -66 + 1.10 x ; Lin’s concordance correlation (95%CI) = 0.8 5 (0.78 – 0.90)
 n = 88; Intercept (SE) = -66 (53), Slope (SE) = 1.1 (0.07)
800 µg/d y = -150 + 1.19 x ; Lin’s concordance correlation (95% CI) = 0.77 (0.67 – 0.84)
 n = 84; Intercept (SE) = -150 (120); Slope (SE) = 1.2 (0.15)
1600
perfect agreement: y = x
1400
1200
1000
Predicted RBC-folate at visit 2 (versus visit 1) , nmol/L
800
600
Predicted 4-weeks RBC-folate levels after recalibration (new intercept and slope) are:
In the 400 µg/d group = 25 + 1.27 * baseline RBC-folate
In the 800 µg/d group = 65 + 1.41 * baseline RBC-folate
400
400
600
800
1000
1200
1400
1600
Measured RBC-folate concentrations (IMMULITE) at visit 2, nmol/L
Supplemental Figure 5. Comparison of measured and predicted RBC-folate using Deming regression and Lin's concordance correlation coefficient after recalibration. RBC, red blood cells; SE, standard error; CI, confidence intervals. The intercepts and slopes are the one obtained after recalibration (see uncorrected regressions in Supplemental Figure 4 for comparison).

## Slide 7
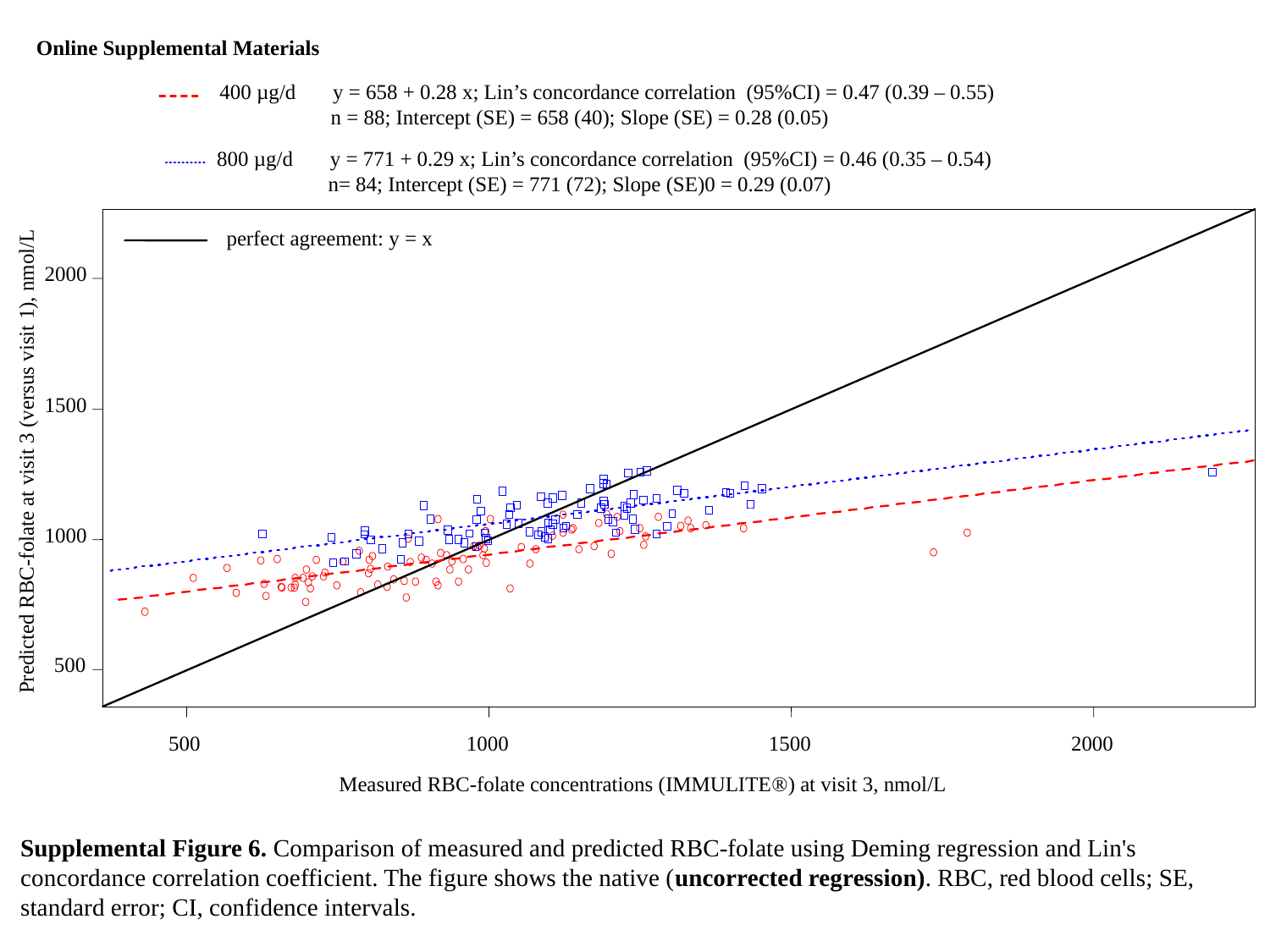

Online Supplemental Materials
400 µg/d y = 658 + 0.28 x; Lin’s concordance correlation (95%CI) = 0.47 (0.39 – 0.55)
 n = 88; Intercept (SE) = 658 (40); Slope (SE) = 0.28 (0.05)
800 µg/d y = 771 + 0.29 x; Lin’s concordance correlation (95%CI) = 0.46 (0.35 – 0.54)
 n= 84; Intercept (SE) = 771 (72); Slope (SE)0 = 0.29 (0.07)
perfect agreement: y = x
2000
1500
Predicted RBC-folate at visit 3 (versus visit 1), nmol/L
1000
500
500
1000
1500
2000
Measured RBC-folate concentrations (IMMULITE) at visit 3, nmol/L
Supplemental Figure 6. Comparison of measured and predicted RBC-folate using Deming regression and Lin's concordance correlation coefficient. The figure shows the native (uncorrected regression). RBC, red blood cells; SE, standard error; CI, confidence intervals.

## Slide 8
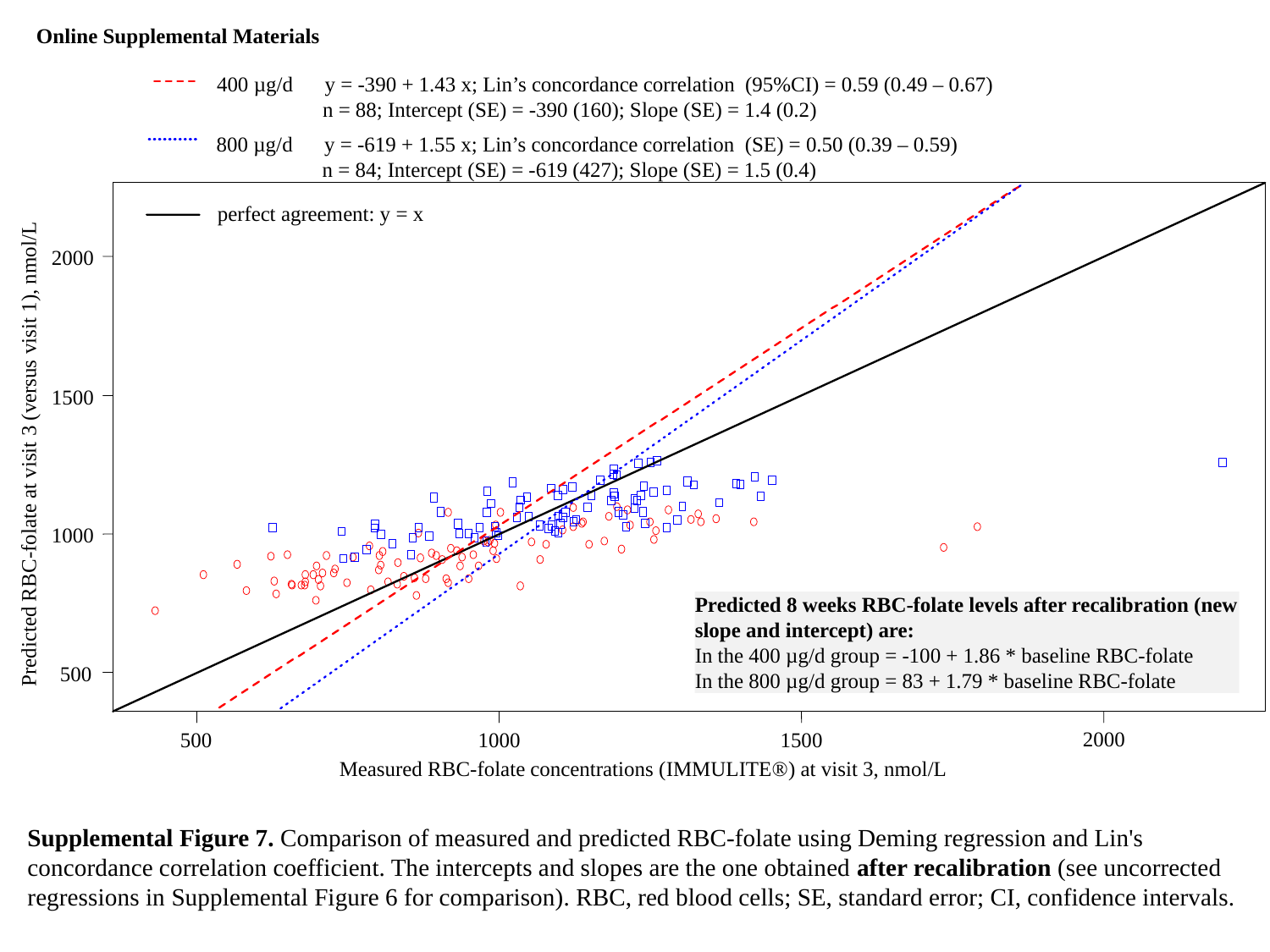

Online Supplemental Materials
400 µg/d y = -390 + 1.43 x; Lin’s concordance correlation (95%CI) = 0.59 (0.49 – 0.67)
 n = 88; Intercept (SE) = -390 (160); Slope (SE) = 1.4 (0.2)
800 µg/d y = -619 + 1.55 x; Lin’s concordance correlation (SE) = 0.50 (0.39 – 0.59)
 n = 84; Intercept (SE) = -619 (427); Slope (SE) = 1.5 (0.4)
perfect agreement: y = x
2000
1500
Predicted RBC-folate at visit 3 (versus visit 1), nmol/L
1000
Predicted 8 weeks RBC-folate levels after recalibration (new slope and intercept) are:
In the 400 µg/d group = -100 + 1.86 * baseline RBC-folate
In the 800 µg/d group = 83 + 1.79 * baseline RBC-folate
500
2000
500
1000
1500
Measured RBC-folate concentrations (IMMULITE) at visit 3, nmol/L
Supplemental Figure 7. Comparison of measured and predicted RBC-folate using Deming regression and Lin's concordance correlation coefficient. The intercepts and slopes are the one obtained after recalibration (see uncorrected regressions in Supplemental Figure 6 for comparison). RBC, red blood cells; SE, standard error; CI, confidence intervals.
