# Supplementary material for: Response of Red Blood Cell Folate to Supplementation in Nonpregnant Women is Predictable: A Proposal for Personalized Supplementation
Source: Mol Nutr Food Res. 2018 Jan 22;62(4):1700537. doi: 10.1002/mnfr.201700537 (PMC5838518; doi:10.1002/mnfr.201700537)
Supplement: Supplementary file 2 — Supplementary material [file MNFR-62-na-s002.docx]

| **Supplemental TABLE 1.** Composition of the multimicronutrient supplements^1,2^. | | |
| --- | --- | --- |
|  | **Supplement I^1^**  **400 µg** | **Supplement II^2^**  **800 µg** |
| Vitamin B-1, mg | 1.4 | 1.2 |
| Vitamin B-2, mg | 1.4 | 1.6 |
| Vitamin B-6, mg | 1.9 | 1.9 |
| Vitamin B-9 (folate), µg | 400 [folic acid,  (6S)-5-CH_3_-H_4_folate-Ca (1:1)] | 800 [folic acid,  (6S)-5-CH_3_-H_4_folate-Ca (1:1)] |
| Vitamin B-12, µg | 2.6 | 3.5 |
| Biotin, µg | 30 | 60 |
| Niacin, mg | 18 | 15 |
| Pantothenic acid, mg | 6 | 6 |
| Vitamin E, mg | 10 | 13 |
| Vitamin C, mg | 85 | 110 |
| Vitamin D, µg (IU) | 5 (200) | 20 (800) |
| Iodine, µg | 150 | 150 |
| Vitamin A, µg | 770 | - |
| Magnesium, mg | 57 | - |
| Iron, mg | 14 | - |
| Copper, mg | 1.0 | - |
| Zinc, mg | 10 | - |
| Omega-3-fatty acids, mg | 200 | - |
| Selenium, µg | 60 | - |

Composition of Multimicronutrient in; ^1^ Elevit®gynvital (capsule); and ^2^ Femibion®1(tablet).

| **Supplemental TABLE 2.** Red blood cells survival fractions. | | | | | |
| --- | --- | --- | --- | --- | --- |
| Days | Mean | 95 % CI | | 2.5 Percentile | 97.5 Percentile |
| 7 | 0.94 | 0.94 | 0.94 | 0.91 | 0.97 |
| 14 | 0.88 | 0.87 | 0.89 | 0.85 | 0.91 |
| 21 | 0.82 | 0.81 | 0.83 | 0.78 | 0.85 |
| **28** | **0.76** | **0.75** | **0.77** | **0.72** | **0.8** |
| 35 | 0.70 | 0.68 | 0.71 | 0.65 | 0.75 |
| 42 | 0.64 | 0.62 | 0.66 | 0.58 | 0.69 |
| 49 | 0.58 | 0.56 | 0.60 | 0.51 | 0.64 |
| **56** | **0.52** | **0.50** | **0.54** | **0.45** | **0.59** |
| 63 | 0.46 | 0.43 | 0.49 | 0.39 | 0.54 |
| 70 | 0.40 | 0.37 | 0.43 | 0.32 | 0.48 |
| 77 | 0.34 | 0.31 | 0.37 | 0.27 | 0.43 |
| **84** | **0.28** | **0.26** | **0.32** | **0.22** | **0.38** |
| 91 | 0.23 | 0.21 | 0.26 | 0.17 | 0.33 |
| 98 | 0.18 | 0.16 | 0.21 | 0.13 | 0.28 |
| 105 | 0.14 | 0.12 | 0.16 | 0.10 | 0.23 |
| 112 | 0.10 | 0.09 | 0.12 | 0.07 | 0.18 |
| 119 | 0.07 | 0.06 | 0.09 | 0.04 | 0.13 |
| 126 | 0.05 | 0.04 | 0.06 | 0.02 | 0.10 |
| 133 | 0.03 | 0.03 | 0.04 | 0.01 | 0.07 |
| The data are re-produced from the original publication by Shrestha et al., (1). The numbers indicate the percentage of RBCs that remain in blood at the indicated time points. | | | | | |

| **Supplemental TABLE 3.** Baseline characteristics of the study participants according to allocation to 400 µg/d or 800 µg/d folate. The study included women of childbearing age who started the trial with a baseline RBC-folate concentration < 906 nmol/L (n = 172). | | | |
| --- | --- | --- | --- |
|  | **Folate dose = 400 µg/d** | **Folate dose = 800 µg/d** | **p^1^** |
| Number | 88 | 84 | **-** |
| Age, years | 26 (23-28) | 25.0 (22.0-28.8) | 0.323 |
| BMI, kg/m^2^ | 21.3 (20.0-23.1) | 21.5 (20.1-22.7) | 0.725 |
| Creatinine, mg/dl | 0.76 (0.71-0.82) | 0.76 (0.71-0.83) | 0.761 |
| ALT, UI | 17 (13-22) | 16 (13-19) | 0.677 |
| AST, UI | 18 (15-21) | 17 (15-20) | 0.409 |
| Haemoglobin, mg/dl | 13.3 (12.7-14.0) | 13.1 (12.4-13.6) | 0.239^2^ |
| Haematocrit, % | 40 (38-41) | 39 (37-41.6) | 0.126^2^ |
| User of hormonal anti-contraceptives, n (%) | 57 (65%) | 51 (61%) | 0.582^3^ |
| Smokers, n (%) | 16 (18%) | 10 (12%) | 0.251^3^ |
| Education, n (%)  University  University student  Less than university | 34 (39%)  30 (34%)  24 (27%) | 30 (36%)  28 (33%)  26 (31%) | 0.858^3^ |
| Data are median (25^th^, 75^th^ percentiles) or n (%).  ^1^ p values are according to Wilcoxon rank sum test (for continuous variables).  ^2^ p values are according to students-t-test.  ^3^ p values are according to Chi-square test. | | | |

| **Supplemental TABLE 4**. Estimated baseline RBC-folate concentrations sufficient to achieve a target level of 906 nmol/L after supplementation with 400 µg/d or 800 µg/d folate for 4 or 8 weeks. | | | |
| --- | --- | --- | --- |
|  |  | **Estimated minimal baseline RBC-folate to achieve at least 906 nmol/L after the specified dose and duration** | |
| Duration | Folate dose | Equations | Starting RBC-folate, nmol/L |
| 4 wks^1^ | 400 µg/d | (906 – 25)/ 1.27 | **694** |
| 4 wks^2^ | 800 µg/d | (906 – 65)/ 1.41 | ***596*** |
| 8 wks^3^ | 400 µg/d | (**694** – 25)/ 1.27 | 527 |
| 8 wks^3^  8 wks^4^ | 800 µg/d  800 µg/d | (***596*** – 65)/ 1.41  (906 – 83)/ 1.79 | 377  460 |
| ^1^RBC-folate (nmol/L) after 4 wks on 400 µg/d = 25 + 1.27 * baseline RBC-folate.  ^2^ RBC-folate (nmol/L) after 4 wks on 800 µg/d = 65 + 1.41 * baseline RBC-folate.  ^3^ The 4 wks equations were applied twice consequently to calculate the starting levels for the 8 wks.  ^4^ The recalibrated 8 weeks equation [predicted RBC-folate = 83 + 1.79 * baseline RBC-folate] was used here to predict baseline RBC-folate necessary to achieve at least 906 nmol/L after 8 weeks on 800 µg/d.  RBC, red blood cell. | | | |

| **Supplemental TABLE 5.**  Prediction of post-intervention RBC-folate based on average baseline RBC-folate data reported in published studies by using the recalibrated models shown in TABLE 3 in the manuscript. | | | | | | |
| --- | --- | --- | --- | --- | --- | --- |
|  |  |  |  | **RBC-folate levels, nmol/L^5^** | | |
| Duration | Dose, µg/d | Prediction equations for post-intervention RBC-folate | Study | Measured  Baseline | Measured  (post) | Calculated  (post) |
| 4 weeks^2^ | 400 | = 25 + 1.27 * baseline RBC-folate | Hao et al., (2) | 625 | 729 | 819 |
|  |  |  | Lamers et al., (3)  Folic acid  5-methylfolate | 668  603 | 840  800 | 873  791 |
|  |  |  | Crider et al., (4)  MTHFR CC  MTHFR CT  MTHFR TT | 718  621  533 | 838  761  659 | 937  814  702 |
| 4 weeks^2^ | 800 | = 65 + 1.41 * baseline RBC-folate | Caudill et al., (5)^1^ | 767 | 970 | 1146 |
| 8 weeks^3^ | 400 | = -100 + 1.86 * baseline RBC-folate | Pietrzik et al., (6) | 706 | 983 | 1213 |
|  |  |  | Pietrzik et al., (6) | 648 | 1038 | 1105 |
|  |  |  | Lamers et al., (3)  Folic acid  5-methylfolate | 668  603 | 950  1025 | 1142  1022 |
| 8 weeks^4^ | 800 | = 83 + 1.79 * baseline RBC-folate | Pietrzik et al., (6) | 654 | 1166 | 1254 |
|  |  |  | Caudill et al., (5)^1^ | 767 | 1130 | 1456 |
| ^1^ RBC-folate were estimated from the figures in Caudill et al., (5).  ^2^ The equations after 4 weeks are recalibrated according to Deming regression to correct for systematic bias.  ^3^ The equation after 8 weeks for the 400 µg/d showed bias despite recalibration.  ^4^ The equation after 8 weeks for the 800 µg/d was corrected for systematic bias by using Deming regression.  ^5^ Folate conversion factor: 1 ng/ml folate = 2.265 nmol/L.  MTHFR; methylene tetrahydrofolate reductase, RBC; red blood cell. | | | | | | |

Reference List

1. Shrestha RP, Horowitz J, Hollot CV, Germain MJ, Widness JA, Mock DM, Veng-Pedersen P, Chait Y. Models for the red blood cell lifespan. J Pharmacokinet Pharmacodyn 2016;43:259-74.

2. Hao L, Yang QH, Li Z, Bailey LB, Zhu JH, Hu DJ, Zhang BL, Erickson JD, Zhang L, Gindler J et al. Folate status and homocysteine response to folic acid doses and withdrawal among young Chinese women in a large-scale randomized double-blind trial. Am J Clin Nutr 2008;88:448-57.

3. Lamers Y, Prinz-Langenohl R, Bramswig S, Pietrzik K. Red blood cell folate concentrations increase more after supplementation with [6S]-5-methyltetrahydrofolate than with folic acid in women of childbearing age. Am J Clin Nutr 2006;84:156-61.

4. Crider KS, Zhu JH, Hao L, Yang QH, Yang TP, Gindler J, Maneval DR, Quinlivan EP, Li Z, Bailey LB et al. MTHFR 677C->T genotype is associated with folate and homocysteine concentrations in a large, population-based, double-blind trial of folic acid supplementation. Am J Clin Nutr 2011;93:1365-72.

5. Caudill MA, Cruz AC, Gregory JF, III, Hutson AD, Bailey LB. Folate status response to controlled folate intake in pregnant women. J Nutr 1997;127:2363-70.

6. Pietrzik K, Lamers Y, Bramswig S, Prinz-Langenohl R. Calculation of red blood cell folate steady state conditions and elimination kinetics after daily supplementation with various folate forms and doses in women of childbearing age. Am J Clin Nutr 2007;86:1414-9.
